# Causal effect estimation in sequencing studies: a Bayesian method to account for confounder adjustment uncertainty

**DOI:** 10.1186/s12919-016-0064-3

**Published:** 2016-10-18

**Authors:** Chi Wang, Jinpeng Liu, David W. Fardo

**Affiliations:** 1 Department of Biostatistics, College of Public Health, University of Kentucky, 725 Rose St, Lexington, KY 40536 USA; 2 Biostatistics and Bioinformatics Shared Resource Facility, Markey Cancer Center, University of Kentucky, 800 Rose St, Lexington, KY 40536 USA

## Abstract

Estimating the causal effect of a single nucleotide variant (SNV) on clinical phenotypes is of interest in many genetic studies. The effect estimation may be confounded by other SNVs as a result of linkage disequilibrium as well as demographic and clinical characteristics. Because a large number of these other variables, which we call potential confounders, are collected, it is challenging to select and adjust for the variables that truly confound the causal effect. The Bayesian adjustment for confounding (BAC) method has been proposed as a general method to estimate the average causal effect in the presence of a large number of potential confounders under the assumption of no unmeasured confounders. In this paper, we explore the application of BAC in genetic studies using Genetic Analysis Workshop 19 exome sequencing data. Our results show that BAC can efficiently estimate the causal effect of genetic variants with adjustment for confounding. Consequently, BAC may serve as a useful tool for genome-wide association studies data analysis to effectively assess the causal effect of genetic variants and the impact of potential interventions.

## Background

In genetic studies, a large number of baseline and genetic variables are observed. The selection and adjustment of these covariates is essential for estimating the average causal effect (ACE). Recently, a method called Bayesian adjustment for confounding (BAC) [1, 2] was proposed to account for the uncertainty in confounder selection while estimating the ACE of a certain exposure variable. BAC uses a Bayesian model averaging (BMA) [3] approach to estimate the ACE by taking a posterior weighted average of ACE estimates from a battery of models with adjustments of different sets of covariates. A key feature of BAC is that it incorporates the strength of associations between covariates in the model and the exposure into the prior for each individual model. This is different from the regular BMA method, which assigns uniform prior weight to each model. It has been shown that large posterior weights in BAC are usually given to models that have fully adjusted for confounding so that an unbiased estimate of ACE can be obtained.

In this paper, we explore the application of BAC in estimating the ACE of single-nucleotide variants (SNVs). Although BAC has been applied to environmental and clinical studies [1, 2], to our knowledge, this is the first time for this method to be applied to genetic studies. We illustrate the application of BAC using Genetic Analysis Workshop 19 (GAW19) sequencing data. Briefly, these data consist of hg19-aligned whole exome sequences from 1943 unrelated Hispanic subjects as part of Type 2 Diabetes Genetic Exploration by Next-generation sequencing in Ethnic Samples (T2D-GENES) Project 1. We focus on evaluating the causal effect of SNVs in *MAP4* and utilize the 200 simulated phenotype sets from these individuals [4].

## Methods

### The causal model

We adopt the Rubin causal model [5, 6] to estimate the ACE of a certain SNV, which we call the exposure, on systolic blood pressure (SBP), which we call the outcome. Let *Y*(*X*) be the potential outcome an individual would have if the genotype was *X*. Here, we assume an additive mode of inheritance so that *X* is the number of alternative alleles, ie, *X* ∈ {0, 1, 2}. The observed outcome *Y* is the outcome associated with an individual’s actual genotype: *Y* = ∑_*x* = 0_^2^ *Y*(*x*)*I*{*X* = *x*}, where *I*{*X* = *x*} is one if the individual’s genotype is *x* or zero otherwise. Thus, the ACE for having one alternative allele is Δ = *E*{*Y*(1)} − *E*{*Y*(0)}. Suppose the true set of confounders, ***U*** ^*^, can be identified. Under the strong ignorability assumption [7], which assumes the potential outcomes and *X* are independent given ***U*** ^*^, *E*{*Y*(*x*)} = *E*{*E*(*Y*|*X* = *x*, U*)}. Therefore, Δ = *E*{*E*(*Y*|*X* = 1, U*)} − *E*{*E*(*Y*|*X* = 0, U*)}. If we further assume a linear regression model for *Y* on *X* and ***U*** ^*^, it can be shown that the ACE is equal to the corresponding model coefficient of *X*.

In practice, however, it is usually uncertain which covariates are true confounders. This is particularly challenging in genetic association studies where many variants are correlated and the true causal variants are unknown. The bias and variance of the ACE estimate can depend strongly on which covariates are included for adjustment in the analysis. To deal with this problem, we propose the following approach.

### The Bayesian adjustment for confounding method

Let ***U*** = {*U*_1_, ⋯, *U*_*M*_} be the set of potential confounders. We assume no unmeasured confounders so that ***U*** ⊇ U*. To adjust for confounders and estimate the ACE, we jointly consider two models: a logistic regression model for the SNV of interest (the exposure model) and a linear regression model for the outcome (the outcome model). Specifically,

1 $$ \log \frac{P\left({X}_i=1\ \mathrm{or}\ 2\left|{\boldsymbol{U}}_i\right.\right)}{P\left({X}_i=0\left|{\boldsymbol{U}}_i\right.\right)}={\delta}_0^{\alpha^X}+{\displaystyle {\sum}_{m=1}^M{\alpha}_m^X{\delta}_m^{\alpha^X}{U}_{im}} $$

2 $$ \mathrm{E}\left\{{Y}_i\Big|{X}_i,{\boldsymbol{U}}_i\right\}={\delta}_0^{\alpha^Y}+{\beta}^{\alpha^Y}{X}_i + {\displaystyle {\sum}_{m=1}^M{\alpha}_m^Y{\delta}_m^{\alpha^Y}{U}_{im}} $$

where *α*_*m*_^*X*^ and *α*_*m*_^*Y*^ are indicators for the inclusion (= 1) or exclusion (= 0) of *U*_*m*_ in the exposure and the outcome models, respectively; *m* indexes SNVs and *i* indexes individuals. For convenience, we refer to parameter vectors ***α*** ^*X*^ = (*α*_1_^*X*^, ⋯, *α*_*M*_^*X*^)^*T*^ and ***α*** ^*Y*^ = (*α*_1_^*Y*^, ⋯, *α*_*M*_^*Y*^)^*T*^ as “models.” For regression coefficients, *β* and *δ*, we use a notation that explicitly keeps track of the fact that these coefficients differ in meaning with the $\boldsymbol{\alpha}$s. Furthermore, to clarify the estimand, it is useful to consider the smallest outcome model that includes all the true confounders. We denote that model by α_*_^Y^. Our estimand, the ACE of *X* on *Y*, is the coefficient of *X* in α_*_^Y^, denoted by β_*_.

As α_*_^Y^ is usually unknown, we use a Bayesian model averaging approach to obtain the posterior of β_*_ by taking a weighted average across the posteriors under each possible model:

3 $$ P\left({\beta}_{*}\left|D\right.\right)={\displaystyle {\sum}_{{\boldsymbol{\alpha}}^Y}p\left({\beta}_{*}\left|{\boldsymbol{\alpha}}^Y\right.,D\right)p\left({\boldsymbol{\alpha}}^Y\Big|D\right)}\approx {\displaystyle {\sum}_{{\boldsymbol{\alpha}}^Y}p\left({\beta}^{\alpha^Y}\left|{\boldsymbol{\alpha}}^Y\right.,D\right)p\left({\boldsymbol{\alpha}}^Y\left|D\right.\right)} $$

where *D* = (***X***, ***Y***, ***U***) denotes the observed data. For a model that contains α_*_^Y^ (meaning that the model includes all the covariates in α_*_^Y^), its model coefficient of *X* is also equal to β_*_. For a model that does not contain α_*_^Y^, its model coefficient of *X* may be different from β_*_. Consequently, the approximation works well if the model weight *p*(***α*** ^*Y*^|*D*) concentrates on models that contain α_*_^Y^. Otherwise, it can be largely biased from the inclusion of models not fully adjusted for confounders.

To ensure large weights are assigned to models that contain α_*_^Y^ and based on the fact that confounders are necessarily associated with both *X* and *Y*, we propose to obtain the posterior of ***α*** ^*Y*^ by $p\left({\boldsymbol{\alpha}}^Y\left|D\right.\right)={\displaystyle {\sum}_{{\boldsymbol{\alpha}}^X}p\left({\boldsymbol{\alpha}}^X,{\boldsymbol{\alpha}}^Y\left|D\right.\right)}$, where the joint posterior of ***α*** ^*X*^ and ***α*** ^*Y*^ is calculated by assuming the following prior:

4 $$ \frac{p\left({\alpha}_m^Y=1\Big|{\alpha}_m^X=1\right)}{p\left({\alpha}_m^Y=0\Big|{\alpha}_m^X=1\right)}=\omega,\ \frac{p\left({\alpha}_m^Y=1\Big|{\alpha}_m^X=0\right)}{p\left({\alpha}_m^Y=0\Big|{\alpha}_m^X=0\right)}=1 $$

5 $$ \frac{p\left({\alpha}_m^X=1\Big|{\alpha}_m^Y=0\right)}{p\left({\alpha}_m^X=0\Big|{\alpha}_m^Y=0\right)}=\frac{1}{\omega },\frac{p\left({\alpha}_m^X=1\Big|{\alpha}_m^Y=1\right)}{p\left({\alpha}_m^X=0\Big|{\alpha}_m^Y=1\right)}=1,m=1,\cdots, M $$

where *ω* ∈ {1, ∞) is a dependence parameter. When $\omega$ > 1, it increases the chance for a covariate associated with *X* to be included into the outcome model. Such a covariate, if also associated with *Y*, is likely to be a confounder. Therefore, the above prior facilitates confounder selection by advocating the use of a covariate’s associations with both the exposure and the outcome to determine its inclusion in or exclusion from the outcome model. It is likely to yield a posterior of ***α*** ^*Y*^ that assigns mass preferentially to models including all the true confounders [2].

In the implementation, we use the MCMCpack package in R to obtain posterior samples of ${\beta}^{\alpha^Y}$ for a given ***α*** ^*Y*^. The posterior samples of ***α*** ^*Y*^ are obtained by using the MC^3^ method [8] where the Bayes factor comparing different outcome models is approximated by a Bayesian information criterion (BIC) approximation [1]. Codes that implement BAC are available at http://sweb.uky.edu/~cwa236/BAC_GAW19.zip.

### Data sets and data filtering

We consider GAW19 sequencing data, which consist of hg19-aligned whole exome sequences from 1943 unrelated Hispanic subjects, as part of T2D-GENES Project 1. The corresponding phenotypic data are from the 200 simulated phenotypic data sets (including the null Q1 trait) generated by GAW19. Because of the lack of age information, 81 subjects are dropped. We focus on the SNVs in the *MAP4* gene as well as those 5 kb up- or downstream of *MAP4*. SNVs that have either zero minor allele frequency (MAF) or low coverage (<20×) are filtered out, which leaves a total of 94 SNVs. Among those SNVs, 25 have true effects on SBP in the simulation model.

## Results

**Fig. 1:**
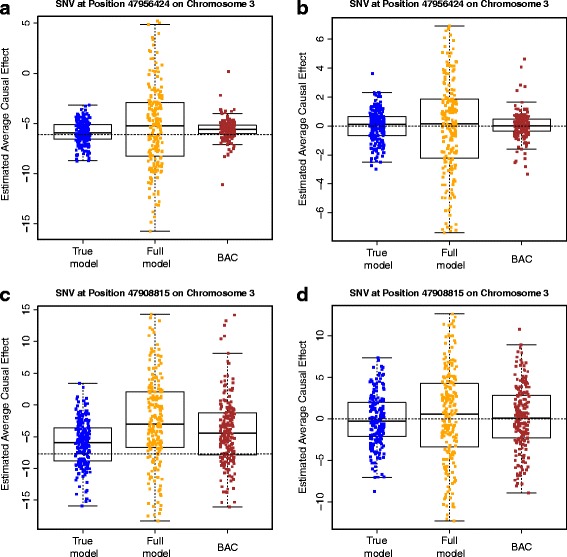
ACE estimates. ACE estimates based on the “true model,” the “full model” and BAC for two *MAP4* SNVs at position 47956424 (**a** and **b**) and position 47908815 (**c** and **d**) on chromosome 3. **a** and **c** are based on 200 simulated phenotypic data sets; **b** and **d** are based on 200 Q1 data sets. The dashed line indicates the true ACE

We evaluated the performance of BAC by using the GAW19 sequencing data after applying the filtering as described in the Methods section. We considered SBP as the outcome and evaluated the estimation of ACE for two SNVs in the *MAP4* gene: 1 common SNV at position 47956424, chromosome 3 (MAF = 0.3435) and one rare SNV at position 47908815, chromosome 3 (MAF = 0.0026). The set of potential confounders include age, sex, their interaction, smoking status, and all the SNVs (other than the SNV of interest) in *MAP4* as well as those 5 kb up- or downstream of *MAP4*. We applied BAC to the set of potential confounders to automatically select and adjust for confounders and to estimate the ACE. We set the dependence parameter ω equal to ten because it appears to provide a good balance between including important confounders and excluding variables only associated with the exposure based on our previous experience. For comparison, we considered the “true model,” which includes age, sex, their interaction, and the 25 SNVs with true effects, and the “full model,” which includes all the potential confounders. Figure 1 and Table 1 summarize the results. In all scenarios, the standard error of ACE estimates based on BAC is smaller than that based on the “full model.” As an example, for the ACE estimation of SNV 47956424 by using simulated phenotypic data, the sample standard error based on BAC is 1.277, which is much smaller than the value 4.280 based on the “full model” and is close to the value 1.121 based on the “true model.” The root mean square error (RMSE) based on BAC is also smaller than that based on the “full model.” Therefore, by performing variable selection and model averaging, BAC is able to effectively reduce the variation and yield a more precise estimate of the ACE.

**Table 1:** Estimation results. Estimation of the ACE on SBP for two *MAP4* SNVs at position 47956424 and position 47908815, chromosome 3

SNV	Data set	Method	BIAS	SEE	SSE	RMSE
47956424 (MAF = 0.3435)	Simulated phenotype	“True model”	0.166	1.206	1.121	1.131
		“Full model”	0.663	4.215	4.280	4.320
		BAC	0.440	1.587	1.277	1.347
	Q1	“True model”	0.006	0.996	1.105	1.102
		“Full model”	0.025	3.483	3.591	3.582
		BAC	0.089	1.313	1.203	1.203
47908815 (MAF = 0.0026)	Simulated phenotype	“True model”	1.617	3.771	3.844	4.161
		“Full model”	5.115	6.89	6.739	8.447
		BAC	3.438	5.322	5.329	6.331
	Q1	“True model”	0.129	3.115	3.113	3.108
		“Full model”	0.369	5.694	5.621	5.619
		BAC	0.179	4.419	3.964	3.958

BIAS is the difference between the mean of estimates of ACE and the true value; RMSE is the root mean square error; SEE is the mean of standard error estimates; SSE is the standard error of the estimates of ACE

Results are based on 200 simulated phenotypic or Q1 data sets. In simulated phenotypic data, the true ACE of SNV at position 47956424 (47908815) is −6.094 (−7.732). In Q1 data, the true ACE of the two SNVs is zero

## Discussion

BAC jointly considers an exposure model and an outcome model, which enables proper selection and adjustment for confounders and yields significantly reduced variation in ACE estimation. For simplicity, the exposure model we consider in the paper is a logistic regression model, where the genotype of the exposure is dichotomized into containing at least one alternative allele or not. One may extend the BAC method by considering a polytomous regression model for the exposure model, where the number of alternative alleles can be taken into account. However, because the exposure model is only used to identify important confounders to be adjusted and the causal effect is estimated based on the outcome model, BAC is relatively robust to the misspecification of the exposure model as long as confounder identification is not largely affected.

The dependence parameter ω indicates the prior strength of connection between the exposure and the outcome models. On the one hand, setting ω equal to one assumes no connection between the two models. Thus, the associations between potential confounders and the exposure will not be accounted for in the variable selection procedure, which may bias the ACE estimation. One the other hand, setting ω equal to ∞ forces all potential confounders that are in the exposure model to be included in the outcome model. Thus, variables that are only associated with the exposure but not with the outcome may be included in the outcome model and inflate the variation of ACE estimation. Therefore, we recommend choosing a finite ω value that can achieve a nice balance between bias and variation. Based on our previous experience, setting ω equal to ten works well in simulation scenarios. A more sophisticated method to determine the optimal ω value can be found in Lefebvre et al. [9].

## Conclusions

The primary goal of genetic association analysis is detection of variants that correlate with some disease phenotype. Replicable associations between variants and many diseases and related endophenotypes have been discovered and subsequently followed with functional studies. While insight into biological function supersedes this primary goal of association study, as substantiated by the era of candidate gene studies, these insights must be pursued for complex disease associations.

The BAC method employed here aims to estimate the causal effect of genetic variants on disease phenotype. These effect metrics represent an attempt to bridge the gap between association and function, while improving the localization of disease-correlated variants. This paper demonstrates that BAC is able to appropriately estimate the causal effect and handle the complexity in the adjustment of confounding as a result of linkage disequilibrium. Our finding that BAC provides a more efficient ACE estimate than conventional methods suggests that BAC has the potential to be widely applied to genome-wide association studies data to effectively assess the causal effect of genetic variants and the impact of potential interventions.
